# A meta-analysis of microarray datasets to identify biological regulatory networks in Alzheimer’s disease

**DOI:** 10.3389/fgene.2023.1225196

**Published:** 2023-08-29

**Authors:** Kimia Sadat Hashemi, Mohadese Koohi Aliabadi, Arian Mehrara, Elham Talebi, Ali Akbar Hemmati, Radin Dabbagh Rezaeiye, Mohammad Javad Ghanbary, Maryam Motealleh, Behnaz Dayeri, Shayan Khalili Alashti

**Affiliations:** ^1^ Department of Genetics, Faculty of Advanced Science and Technology, Tehran Medical Sciences, Islamic Azad University, Tehran, Iran; ^2^ Faculty of Interdisciplinary Science and Technology, Tarbiat Modares University, Tehran, Iran; ^3^ School of Pharmacy, Ramsar International Campus, Mazandaran University of Medical Sciences, Ramsar, Iran; ^4^ Department of Biology and Biotechnology, Molecular Biology, and Genetics, Pavia University, Lombardi, Italy; ^5^ Faculty of Basic Sciences, Gonbad Kavous University, Gonbad Kavous, Iran; ^6^ Institute of Biochemistry and Biophysics, University of Tehran, Tehran, Iran; ^7^ Department of System Biology Lab, University of Vrije Universiteit Amsterdam, Amsterdam, Netherlands; ^8^ Department of Pharmaceutical Sciences, Faculty of Pharmaceutical Biotechnology, University of Milan, Milan, Italy; ^9^ Epilepsy Research Center, Shiraz University of Medical Sciences, Shiraz, Iran

**Keywords:** Alzheimer’s disease, gene expression profiling, microarray analysis, bioinformatics, biology

## Abstract

**Background:** Alzheimer’s Disease (AD) is an age-related progressive neurodegenerative disorder characterized by mental deterioration, memory deficit, and multiple cognitive abnormalities, with an overall prevalence of ∼2% among industrialized countries. Although a proper diagnosis is not yet available, identification of miRNAs and mRNAs could offer valuable insights into the molecular pathways underlying AD’s prognosis.

**Method:** This study aims to utilize microarray bioinformatic analysis to identify potential biomarkers of AD, by analyzing six microarray datasets (GSE4757, GSE5281, GSE16759, GSE28146, GSE12685, and GSE1297) of AD patients, and control groups. Furthermore, this study conducted gene ontology, pathways analysis, and protein-protein interaction network to reveal major pathways linked to probable biological events. The datasets were meta-analyzed using bioinformatics tools, to identify significant differentially expressed genes (DEGs) and hub genes and their targeted miRNAs’.

**Results:** According to the findings, *CXCR4*, *TGFB1*, *ITGB1*, *MYH11*, and *SELE* genes were identified as hub genes in this study*.* The analysis of DEGs using GO (gene ontology) revealed that these genes were significantly enriched in actin cytoskeleton regulation, ECM-receptor interaction, and hypertrophic cardiomyopathy. Eventually, *hsa-mir-122-5p, hsa-mir-106a-5p, hsa-mir-27a-3p, hsa-mir16-5p, hsa-mir-145-5p, hsa-mir-12-5p, hsa-mir-128-3p, hsa-mir 3200-3p, hsa-mir-103a-3p*, and *hsa-mir-9-3p* exhibited significant interactions with most of the hub genes.

**Conclusion:** Overall, these genes can be considered as pivotal biomarkers for diagnosing the pathogenesis and molecular functions of AD.

## 1 Introduction

Alzheimer’s disease (AD) is defined as an age-related progressive neurodegenerative disorder that causes permanent damage to memory cells and cognitive dysfunctions, leading to dementia (Bondi et al., 2017). In terms of clinical symptoms, AD is associated with mental deterioration, memory deficit, delusion, and other cognitive alterations such as early loss of prospective memory. Eventually, in the advanced stages of Alzheimer’s disease, more severe ramifications such as cardiac failure can result in mortality (Ghazal et al., 2022). This disease currently disturbs ∼2% of the people in industrialized countries and its prevalence is predicted to rise dramatically over the next 40 years (Vaz and Silvestre, 2020).

Pathologically, hyperphosphorylation of tau protein and extracellular deposits of beta-Amyloid peptides as plaques, resulting in the formation of neurofibrillary tangles and loss of neuronal cells, constitute the microscopic features of AD (Verheijen and Sleegers, 2018). Despite making significant progress in order to clarify important aspects of AD, a proper diagnosis is not yet available. However, early diagnosis through magnetic resonance imaging (MRI), Position Emission Tomography (PET), and functional MRI (fMRI) of the brain can improve a patient’s life with appropriate treatment and symptom alleviation (Long and Holtzman, 2019).

Although existing treatments remain mostly limited, advancements have been made in defining genetic factors related to the development of Alzheimer’s disease (Rao et al., 2014). Mutations in *APP* (OMIM: 104760, 21q21), *PSEN1* (OMIM: 104311, 14q24), *PSEN2* (OMIM: 600759, 1q42), and *APOE* (OMIM:107741, 19q13) which mostly follow the pattern of autosomal dominant inheritance are known to have substantial impact on AD’s vulnerability (Avramopoulos, 2009). The *APOE* gene which encodes apolipoprotein E, provides a critical link between the central nervous system (CNS) and the periphery, leading to AD by disrupting the integrity of the blood-brain barrier (Husain et al., 2021). In addition, *PSEN1* and *PSEN2*, which encode Presenilin-1 and Presenilin-2, in turn, play a crucial role in memory maintenance and even in the process of beta-amyloid generation (Dai et al., 2018). Consequently, Mutation in these genes may lead to alterations in beta-amyloid formation and the accumulation of these precursors converts beta-amyloid into plaques, leading to the progression of AD (Xiao et al., 2021).

MicroRNAs are a class of small, non-coding RNAs that play a pivotal role in regulating gene expression by either inducing mRNA degradation or suppressing translation (Xiao et al., 2021). They are also responsive to microenvironmental stressors and are involved in maintaining homeostasis, as well as regulating various processes such as cell proliferation, differentiation, and neurodegenerative procedures (Kumar and Reddy, 2016). Given their multi-system and overlapping regulatory roles in critical areas such Cerebral Neocortex, limbic system, and central nervous system, microRNAs found in blood, cerebrospinal fluid (CSF), or brain tissue are presented as promising biomarkers for AD, as alterations in particular miRNAs have been linked to the development of progressive neurological disorders such as AD (Delay et al., 2011; Wang et al., 2019).

The primary objective of this study is to examine the involvement of miRNAs and their target genes in the context of Alzheimer’s disease, through a bioinformatic meta-analysis approach. Therefore, we identified differentially expressed genes (DEGs) between AD cases and healthy controls, as well as their protein-protein interactions and signaling pathways. By revealing the molecular mechanisms underlying AD, this study could not only pave the way for the development of more effective treatments for this neurodegenerative disorder but also highlight the potential of these pathways as therapeutic targets.

## 2 Materials and methods

### 2.1 Data sources and selection of eligible gene expression datasets for meta-analysis

**FIGURE 1:**
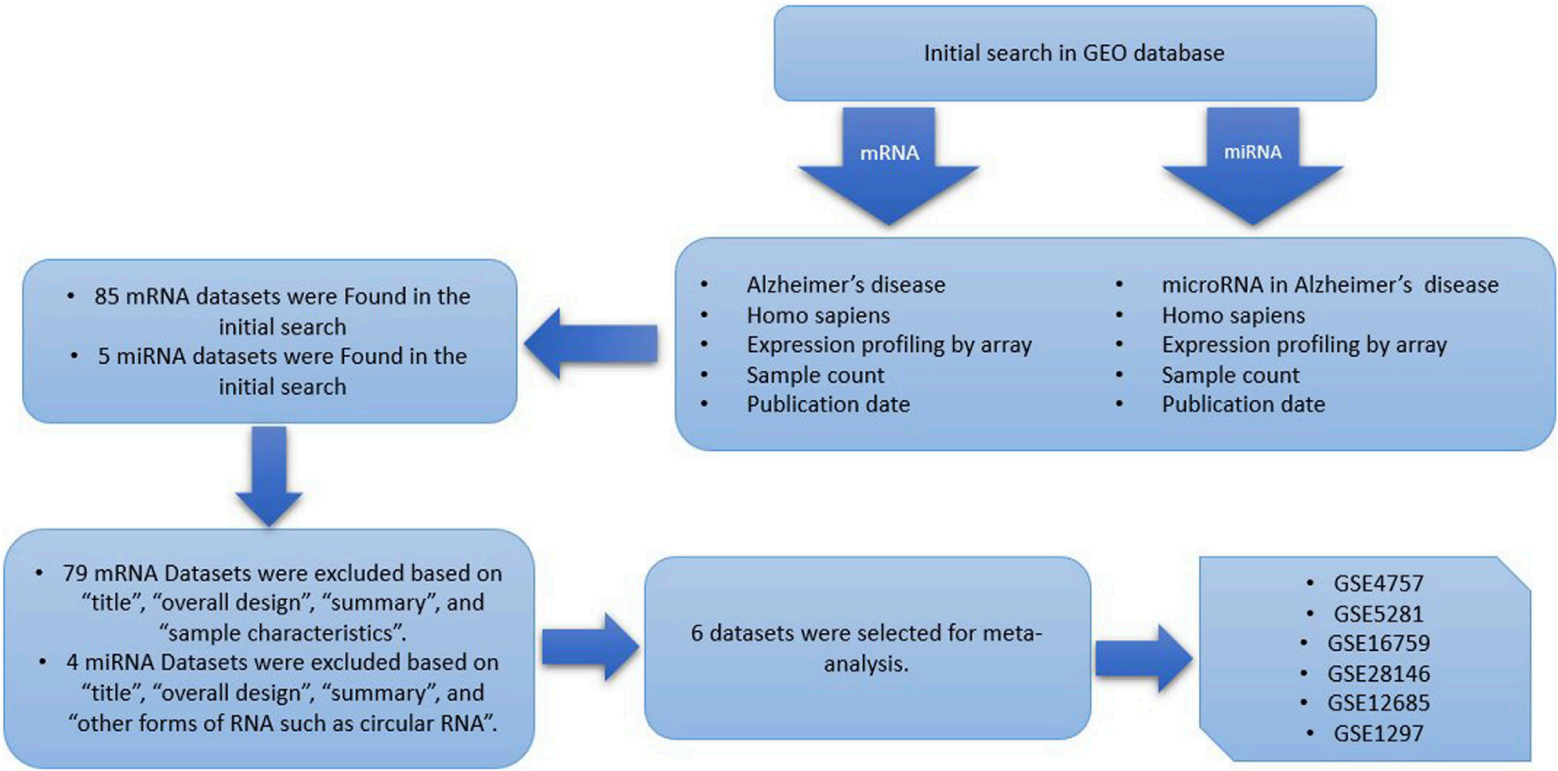
Data set selection flow chart showed that a total of 85 data sets from GEO were evaluated. Finally, 6 data sets for mRNA and a single dataset for miRNA were selected to be included in this meta-analysis.

In this study, multiple independent datasets were used to identify differentially expressed genes and microRNA’s when comparing Alzheimer patients with healthy controls, by performing cross-study meta-analysis research. The research data in this paper, including the expression profile data sets of Microarray, were retrieved from the Gene Expression Omnibus (GEO, https://www.ncbi.nlm.nih.gov/gds/) database with “Alzheimer’s disease,” “*homo sapiens,*” and “expression profiling by array” as keywords. This inclusive set of criteria should be followed when selecting qualified studies and datasets (Bondi et al., 2017): Human case-control studies (Ghazal et al., 2022); gene expression profiling analysis (Vaz and Silvestre, 2020); comparable, untreated test conditions (Verheijen and Sleegers, 2018); available complete raw and processed microarray data. Other clinical covariates, including age, sex, and therapeutic status were not available for all samples, therefore, to avoid the introduction of false positives by imputation, have not been included. Studies were disqualified if they met any of the following criteria (Bondi et al., 2017): letters, abstracts, meta-analyses, review articles, and human case reports (Ghazal et al., 2022); cell lines used in experimental design (Vaz and Silvestre, 2020); RT-PCR used only for profiling studies (Verheijen and Sleegers, 2018); studies without case-control (Long and Holtzman, 2019); studies that examined the impact of specific factors on Alzheimer disease; and (Rao et al., 2014) studies including other forms of RNA such as circular RNA. The datasets and references that met the aforementioned requirements were all manually reviewed. For selecting eligible datasets, the entire pipeline is depicted in Figure 1.

### 2.2 Data extraction and processing

For each dataset, the series matrix file was downloaded and processed in several steps including background correction, log2 transformation, and quantile normalization which were performed using R language v4.2.2. We used the R packages including, Limma, GEOquery, BiocGenerics, Biobase, parallel, reshape, reshape2, ggplot2, grid, plyr, dplyr, data.table, sva, and affy from Bioconductor to process the data from Illumina platforms. After computation, all Alzheimer data sets were merged according to Hughey and Butte’s previously published pipeline (Zetterberg et al., 2004). During this step, the expression data were mean-centered and reduced to the number of common probes across all data sets. The gene expression data of the merged datasets were batch-adjusted using the ComBat method, implemented in the *sva* package v3. Assessing the success of batch correction was confirmed by boxplots.

### 2.3 Differential gene expression

The meta-analysis approach was utilized to evaluate the differential expression of genes between Alzheimer’s disease patients and controls. This analysis was performed using R packages, applying a significance threshold of a false discovery rate (FDR (, *p*-value less than 0.05 and a logarithm fold change (LogFC) of 1.23 or greater. We generated heatmaps using Python version 3.11 to visually represent the data.

### 2.4 Differential miRNA expression

Differentially expressed miRNAs between AD patients and controls were determined by using R packages, using a cutoff false discovery rate (FDR), *p*-value of less than 0.05 and logarithm fold change (LogFC) of 2 or more.

### 2.5 Enriched gene ontology and pathways analysis

Gene ontology (GO) analysis is a widely used method for annotating and enriching functional information of the genes which is categorized into three sections: Biological process (BP), Molecular function (MF), and Cellular component (CC). In this study, GO enrichment and Kyoto Encyclopedia of Genes and Genomes (KEGG) pathway analyses of DEGs were performed using the Enrich database (https://maayanlab.cloud/Enrichr/). To establish statistical significance, certain criteria were employed, with the requirement of at least three genes in a cluster, a GO tree interval range between 3 and 8, and a kappa score of 0.4 for pathway network connectivity. Furthermore, a *p*-value less than 0.05 was deemed significant according to these criteria.

### 2.6 Clustering gene expression data

Python libraries including matplotlib, and seaborn were imported to cluster DEGs and visualize the results. In this section of our research, we used hierarchical clustering, a potent technique for examining high throughput expression data. Python determined the degree of similarity among the genes in each set of data, displayed the expression value using colors, and then clustered the genes.

### 2.7 Protein-protein interaction (PPI) network construction, cluster networks, and identification of hub genes

The possible interactions among DEGs were explored and visualized using the STRING (search tools for the retrieval of interacting Genes/Proteins) darabase (https://string-db.org). The STRING database aims to provide a critical assessment and integration of protein-protein interaction, including direct (physical) as well as indirect (functional) associations. The PPI network was visualized by the Cytoscape V3.9.1 software (https://www.cytoscape.org). Generally, hub genes are defined as genes with high correlation in candidate modules. This study utilized certain criteria as cut-off thresholds for selecting hub genes, including genes with Degree>100, k score>2, and max depth>100. Cytohubba was used to determine the top 10 potential hub genes, also referred to as common top 10 nodes, based on their degree, closeness, and betweenness. Moreover, the Molecular Complex Detection (MCODE) tool was utilized to visualize clusters of PPI networks with specific parameters including, degree cut-off of 2, node score cut-off of 0.2, k score of 2, and max depth of 100. By using the web tool available at (https://bioinformatics.psb.ugent.be/webtools/Venn), a Venn diagram was illustrated to determine the shared hub genes.

### 2.8 Hub genes validation

We used receiver operating characteristic (ROC) curve analysis to assess the predictive impact of hub genes on the risk of AD. The area under the ROC curve was computed to compare the diagnostic efficacy of the hub genes. The analysis was conducted using v.3.11 of the Python packages.

### 2.9 Evaluation of miRNA-hub genes interaction network

To identify the targeted miRNAs of the hub genes, the miRTarBase database (https://www.mirtarbase.cuhk.edu.cn), mirWalk (https://mirwalk.umm.uni-heidelberg.de/), and TargetScan (https://www.targetscan.org/) were used. In addition, the miRNet database (https://www.mirnet.ca/) was used to create a visual representation of the interactions between miRNAs and hub genes.

## 3 Results

### 3.1 Characteristics of datasets for analysis

Our pre-specified criteria led to the identification of six datasets, namely, GSE4757, GSE5281, GSE28146, and GSE16759 from GPL570 (Affymetrix Human Genome U133 Plus 2.0 Array), consisting of 123 samples from AD patients and 96 samples from healthy controls. Additionally, we identified GSE12685, and GSE1297 from GPL96 (Affymetrix Human Genome U133A Array), comprising 29 AD samples and 16 normal samples. In terms of miRNA datasets, GSE16759 (USC/XJZ Human 0.9 K miRNA-940-v1.0) was the only qualified one for further analysis. Furthermore, the microarray data analysis utilized different sample sources, including the brain’s entorhinal cortex, Hippocampal CA1 tissue, and frontal cortex Synaptoneurosome. Table 1 represents the essential features of the datasets that were incorporated.

**TABLE 1:** Characteristics of each selected microarray dataset for the meta-analysis.

GEO accession number	Sample (Normal/AD)	Sample source	Platform
mRNA			
GSE4757	20 (10/10)	Entorhinal cortex	GPL570
GSE5281	161 (74/87)	Entorhinal cortex, Hippocampus, Medial Temporal Gyrus, posterior Cingulate, Superior Frontal Gyrus, primary visual cortex	GPL570
GSE16759	8 (4/4)	Parietal lobe	GPL570
GSE28146	30 (8/22)	CA1 tissue gray matter	GPL570
GSE12685	14 (8/6)	Frontal cortex synaptoneurosome	GPL96
GSE1297	31 (9/22)	Hippocampal CA1 tissue	GPL96
miRNA			
GSE 16759	8 (4/4)	Parietal lobe	GPL8757

### 3.2 Identification of common DEGs and microRNAs in AD

**FIGURE 2:**
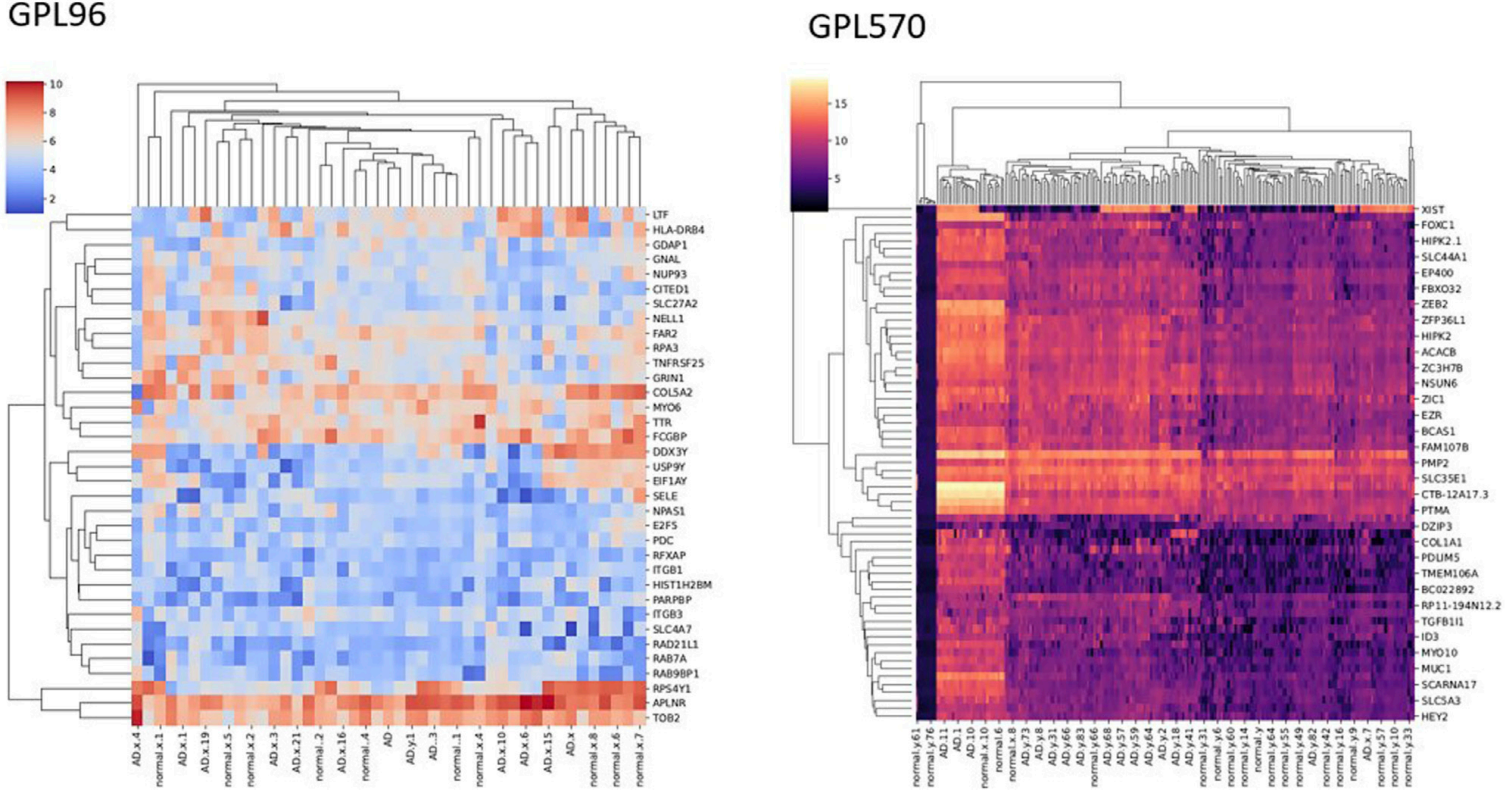
Heatmap of the DEGs with significant LogFC.

**FIGURE 3:**
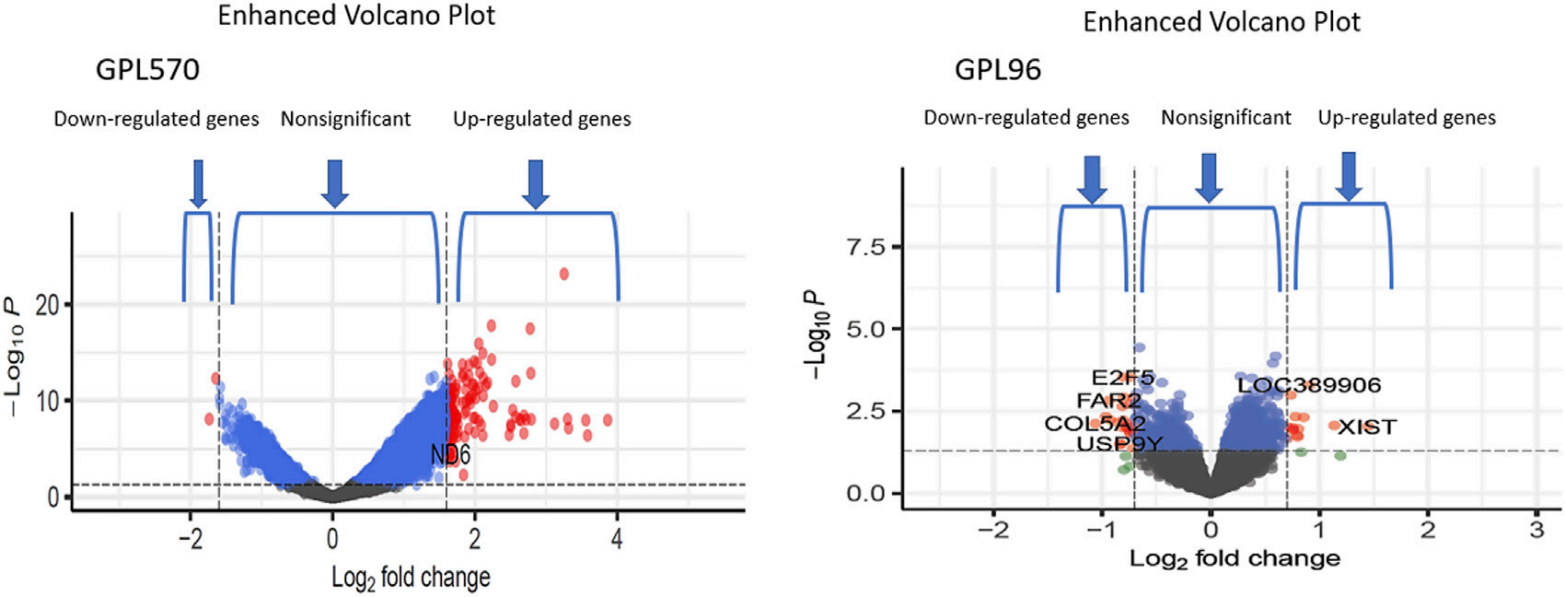
The volcano plots show the upregulated and downregulated differentially expressed genes of the Alzheimer and normal groups. The *x*-axis represents the log 2-fold change (FC), and the *y*-axis represents log 10 (*p* values).

We achieved the result by analyzing six microarray datasets obtained from two distinct sets of microarray platforms (GPL570, GPL96). The outcome of our mRNA analysis revealed a total of 100 differentially expressed genes, of which 73 were upregulated and 27 were downregulated (Figure 2). These DEGs are represented in improved volcano plots that display the DEGs across all samples (Figure 3). Table 2 provides a classification of the upregulated and downregulated genes that were commonly identified as highly significant in the micro array meta-analysis. Among the 73 upregulated genes, *MALAT1* displayed the highest log (FC) with the value of 2.25. On the other hand, *SST* was identified as having the lowest log (FC) value of −1.73 among the 27 downregulated genes.

**TABLE 2:** Expressional profiles for top up- and downregulated DEGs identified by meta-analysis. Were ranked by combined Log (FC) and *p*. value.

Upregulated			Downregulated		
Genes	Log (FC)	*P*. value	Genes	Log (FC)	P.value
*MALAT1*	2.257117	1.80*10 ^ -7	*SST*	−1.73726	8.03*10 ^ -9
*LIFR*	2.11362	1.32*10 ^ -9	*DZIP3*	−1.64435	4.89*10 ^ -13
*COL1A1*	1.997217	1.45*10 ^ -5	*NDUFA7*	−1.58031	4.08*10 ^ -12
*HIPK2*	1.962706	2.38*10 ^ -12	*COL5A2*	−1.06153	0.007384
*CXCR4*	1.934889	1.80*10 ^ -10	*FAR2*	−0.93558	0.001476
*XIST*	1.837898	0.005052	*GRIN1*	−0.92876	0.005726
*ITGB8*	1.754946	4.72*10 ^ -7	*TTR*	−0.85573	0.006913
*TGFB1*	1.666153	6.67*10 ^ -6	*SELE*	−0.83317	0.032153
*MYO6*	0.751279	0.009892	*RPA3*	−0.81267	0.002207
*ITGB3*	0.700192	0.021208	*E2F5*	−0.80603	0.00029

**FIGURE 4:**
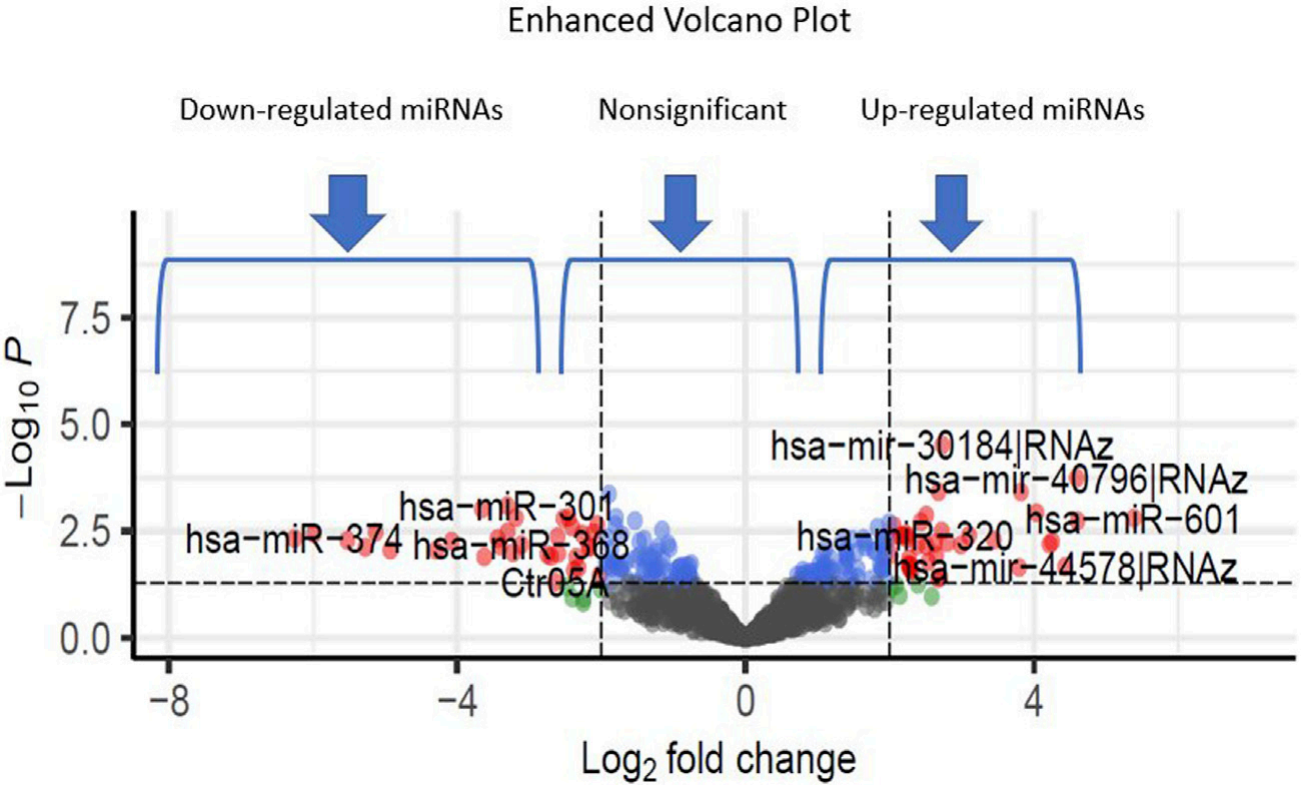
The volcano plots show the upregulated and downregulated miRNAs of the Alzheimer and normal groups. The *x*-axis represents the log 2-fold change (FC), and the *y*-axis represents log 10 (*p* values).

Additionally, through analyzing a single miRNA dataset with the platform of GPL8757, 73 miRNAs were identified as top ones, involved in Alzheimer’s disease, based on their *p*-value and LogFC. *hsa-miR-601* and *hsa-miR-374* were found to be the most significant upregulated and downregulated miRNAs among all, respectively Figure 4.

Table 3. provides a classification of the upregulated and downregulated miRNAs that were commonly identified as highly significant in the micro array meta-analysis.

**TABLE 3:** Expressional profiles for top up- and downregulated miRNAs identified by meta-analysis. Were ranked by combined Log (FC) and *p*. value.

Upregulated			Downregulated		
ID	Log (FC)	P.value	ID	Log (FC)	P.value
*hsa-miR-601*	5.389203	0.001562	*hsa-miR-374*	−6.25921	0.004687
*hsa-mir-23974|RNAz*	4.604908	0.001794	*hsa-miR-582*	−5.98935	0.003615
*hsa-mir-40796|RNAz*	4.592553	0.00018	*hsa-mir-05109|RNAz*	−5.52592	0.005163
*hsa-mir-44578|RNAz*	4.43394	0.021127	*hsa-mir-12504|RNAz*	−5.27599	0.007518
*hsa-miR-575*	4.254831	0.00554	*hsa-mir-12497|RNAz*	−5.13248	0.003349
*hsa-miR-765*	4.215826	0.006291	*hsa-mir-40321|RNAz*	−4.92932	0.008768
*hsa-mir-06383|RNAz*	4.037764	0.00117	*hsa-miR-380-3p*	−4.29229	0.008855
*hsa-miR-188*	3.819711	0.00038	*hsa-miR-30e-5p*	−4.07881	0.005431
*hsa-mir-35582|RNAz*	3.79497	0.022237	*hsa-miR-424*	−3.65906	0.000958
*hsa-miR-671*	3.101654	0.003987	*hsa-miR-153*	−3.62604	0.012613

### 3.3 Gene ontology (GO) enrichment and pathways analysis

**FIGURE 5:**
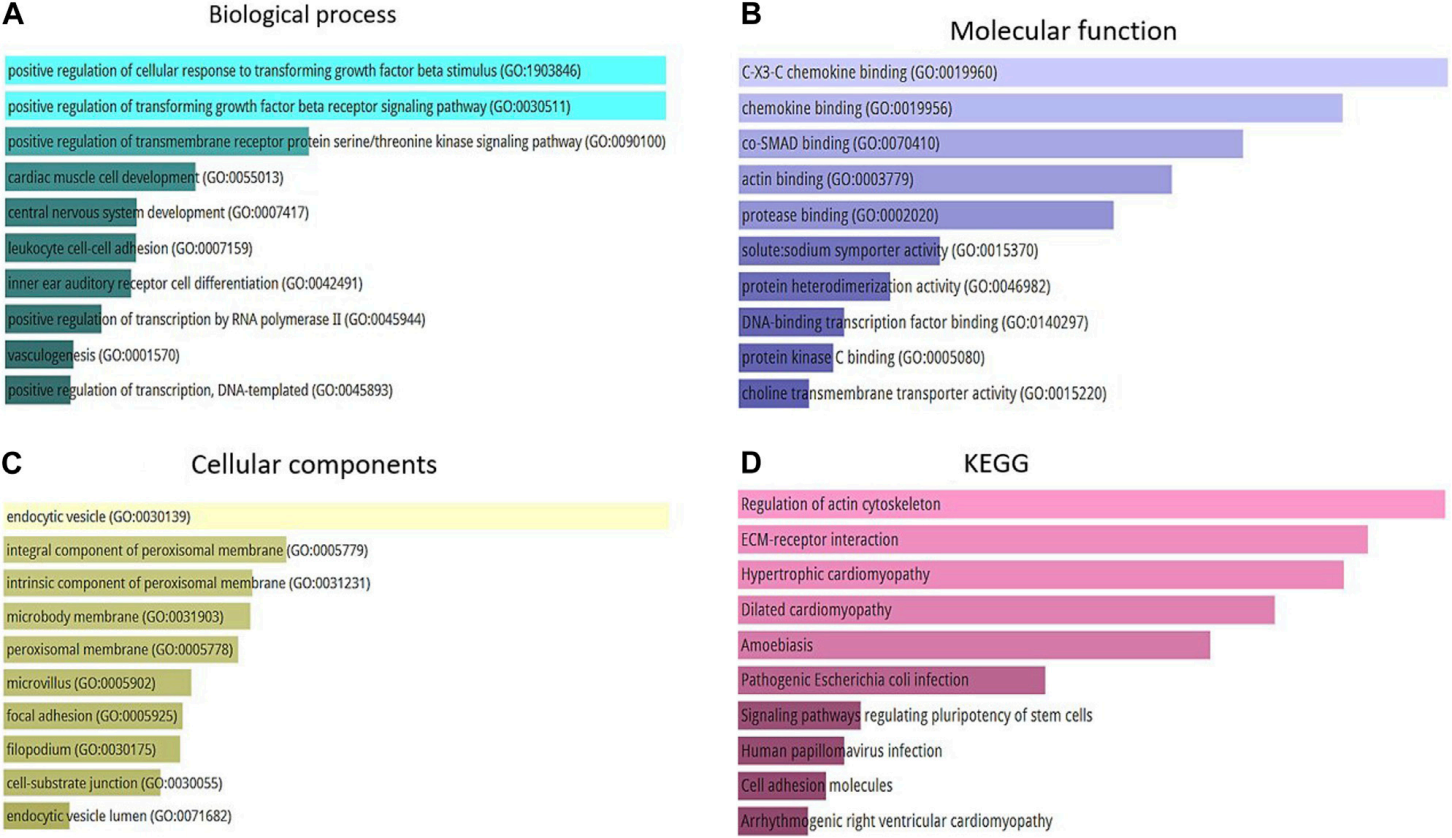
**(A)** Top 10 of biological process. **(B)** Top 10 of molecular function. **(C)** Top 10 of a cellular component. **(D)** Top 10 of KEGG pathway enrichment.

The present study conducted a Gene Ontology enrichment analysis of differentially expressed genes (DEGs) to identify the underlying biological pathways of Alzheimer’s diseases. The results demonstrated that in terms of biological processes (BP), the DEGs were significantly enriched in positive regulation of cellular response to transforming growth factor beta stimulus (GO:1903846), positive regulation of transforming growth factor beta receptor signaling pathway (GO:0030511), and positive regulation of transmembrane receptor protein serine/threonine kinase pathway (GO:0090100) (Figure 5A). For molecular function, the DEGs were mainly enriched in C-X3-C chemokine binding (GO:0019960), chemokine binding (GO:0019956), and co-SMAD binding (GO:0070410) (Figure 5B). As for cellular components the result revealed that the DEGs were mainly enriched in endocytic vesicle (GO:0030139), integral component of proximal membrane (GO:0005779), and intrinsic component of proximal membrane (GO:0031231 (Figure 5C). Furthermore, the KEGG analysis results indicated that these DEGs were enriched in several pathways, including regulation of actin cytoskeleton, ECM-receptor interaction, and hypertrophic cardiomyopathy (Figure 5D). Accordingly, the GO analysis of DEGs indicated their critical enrichment in signaling pathways, including TGFβ signaling, which its dysregulation has been associated with neurodegenerative and cognitive impairment, including Alzheimer’s disease. It is worth mentioning that endocytic vesicle dysfunction, which its critical role has been demonstrated in KEGGs, can lead to abnormal accumulation of tau protein which is a substantial hallmark of Alzheimer’s disease.

### 3.4 PPI network construction and hub genes extraction

**FIGURE 6:**
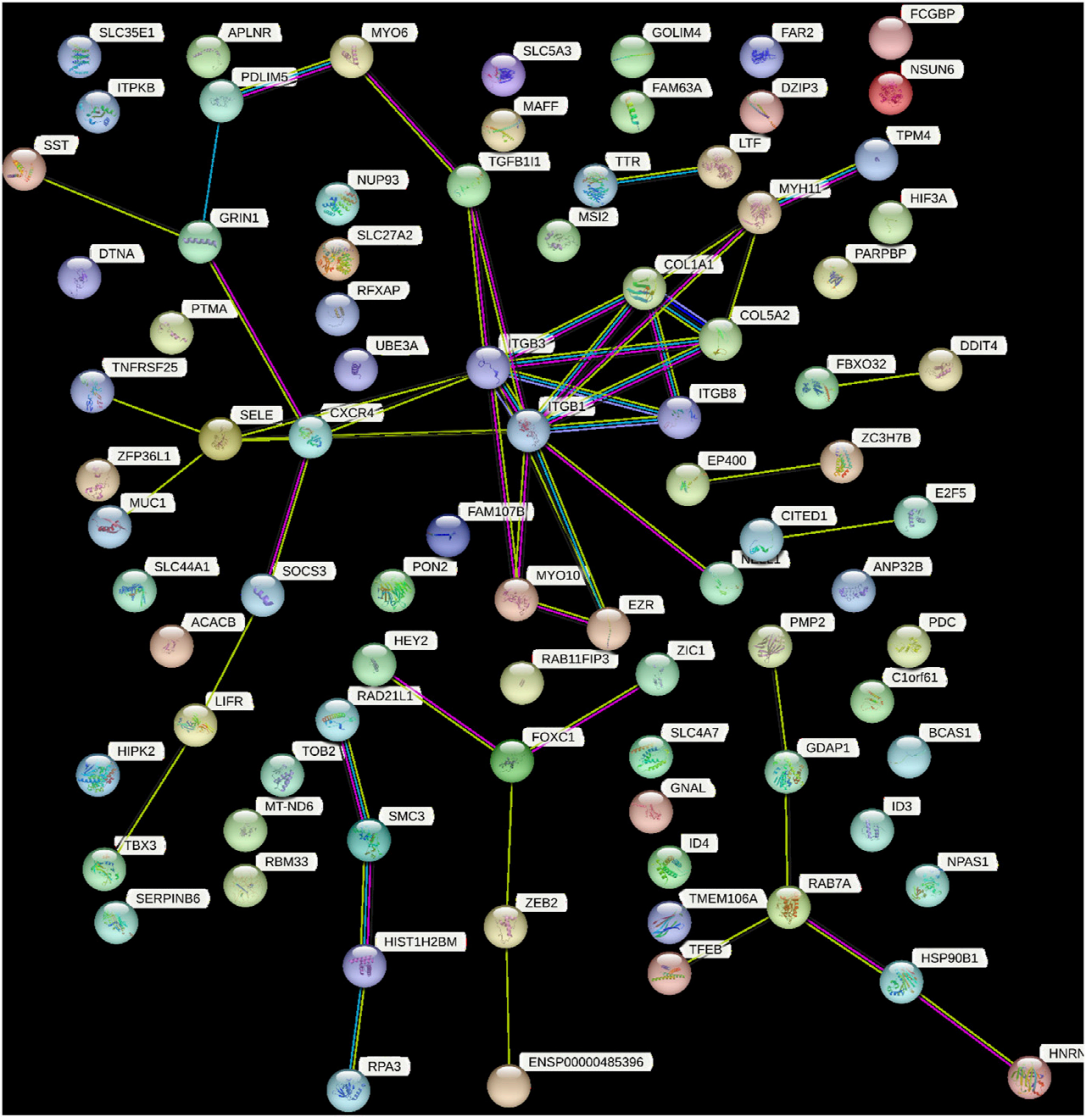
This figure demonstrated the network of predicted associations for a particular group of proteins. The network nodes are proteins and the edges represent the predicted functional associations. Colored lines between the proteins indicate the various types of interaction evidence. The thickness of the line indicated the degree of confidence prediction of the interaction. The other proteins with no associations to other protein in the network were removed.

PPI networks are mathematical representations of the physical contacts between proteins in the cell which play a crucial role in various fundamental molecular mechanisms in living cells. The PPI networks of 100 DEGs included 86 nodes connected by 52 edges with an average clustering coefficient of >0.4 (Figure 6). To determine whether the number of connections identified in our PPI analysis is higher than random chance, we conducted a thorough statistical analysis. Specifically, we performed a simulation-based approach to establish the background distribution of the number of connections (edges) that would occur by random chance. This was achieved by simulating 1,000 sets of randomly selected 100 genes from our dataset, and for each set, we calculated the number of connections. The empirical *p*-value was calculated as the proportion of simulations in which the number of connections equaled or exceeded the observed 52 edges. This *p*-value provides a measure of the statistical significance of our finding.

Our results indicate that the observed number of connections in the PPI network is significantly higher than what would be expected by chance (empirical *p*-value <0.05). This suggests that the interactions among the 100 DEGs are not random and likely reflect biologically meaningful relationships.

**FIGURE 7:**
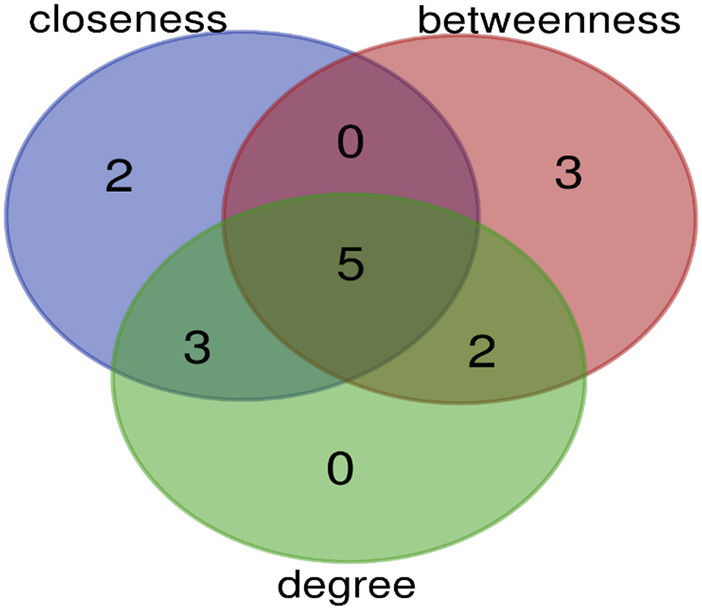
The overlap of hub genes between three methods (degree, closeness, and betweenness) is shown by Venn diagram. Five genes are common between these methods.

The top 10 hub genes were listed in Table 2. The findings indicated that the hub genes identified through three different methods (Degree, closeness, and betweenness), were *CXCR*_*4,*_ *SELE, ITGβ1, MYH11,* and *TGFβ1* (Figure 7).

### 3.5 Molecular complex detection (MCODE) cluster for identification of hub genes

**FIGURE 8:**
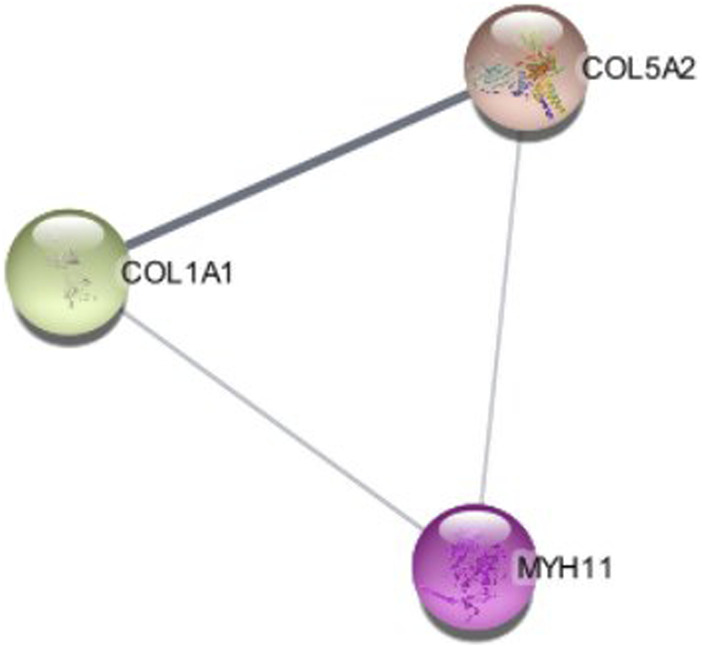
The cluster modules extracted by MCODE.

Significant modules of the PPI network were determined by MCODE. According to this, MCODE score of >5 was set as a significant threshold. The gene *MYH11* in this cluster overlapped with CytoHubba network hub genes, that simultaneously have strong connectivity in the STRING network (Figure 8).

### 3.6 Validation of hub genes

By utilizing the ROC curve to assess the prognostic value of hub genes in the PPI network, 3 out of 5 shared hub genes, namely, *CXCR4, MYH11,* and *TGFB1,* showed potential indications as potential biomarkers for Alzheimer’s disease. However, the remaining two genes (*SELE* and *ITGB1),* were not validated, probably owing to the limited number of samples retrieved from GPL96. To ensure the accuracy and reliability of these genes as potential biomarkers, conducting further investigations using larger sample groups is highly recommended.

**FIGURE 9:**
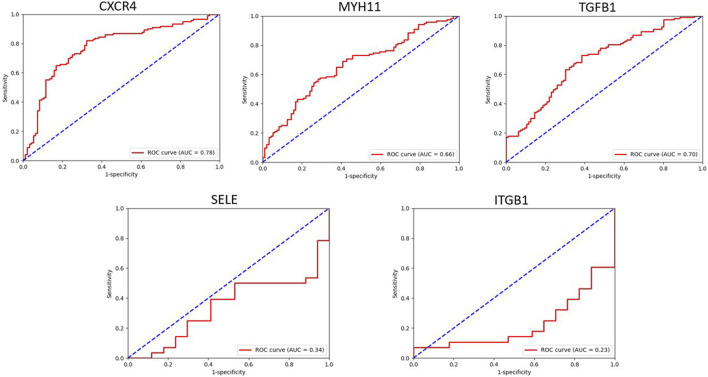
This figure showed the evaluation of sensitivity and specificity of hub genes in the diagnostic of AD.

The area under the ROC curve (AUC) reflects diagnostic value of the test. Accordingly, for these genes, AUC values varied between 0.2 and 0.7 (Figure 9).

### 3.7 Identification of miRNA-hub genes interaction

**FIGURE 10:**
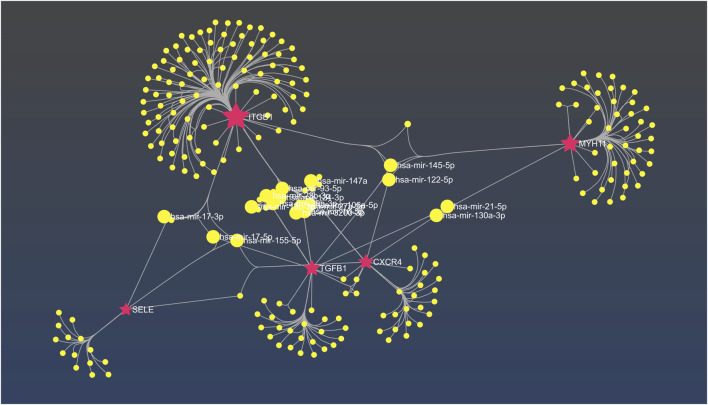
The interaction network between hub genes and miRNAs in miRNet. The fuchsia nodes represent hub genes and the yellow nodes are hsa miRNAs.

Based on the result obtained from miRNet tool, *hsa-mir-122-5p, hsa-mir-106a-5p, hsa-mir-27a-3p, hsa-mir16-5p, hsa-mir-145-5p, hsa-mir-12-5p, hsa-mir-128-3p, hsa-mir 3200-3p, hsa-mir-103a-3p*, and *hsa-mir-9-3p* exhibit considerable interaction with the majority hub genes which is determined as principal miRNAs in the commencement and development of AD (Figure 10).

## 4 Discussion

This meta-analysis aimed to reveal the potential biomarkers of AD, which is known as a progressive neurodegenerative disorder, characterized by the accumulation of amyloid beta and neurofibrillary tangles of hyperphosphorylated tau proteins. In November 1901 Auguste D, presented a long-term study of the female patient and then in 1906, Alois Alzheimer identified and characterized the linkage between histological changes that became known as plaques to the dementia. In the early 1980s, the term “AD” was used to describe cognitive impairment caused by plaques and neurofibrillary tangles. In 1984, Stroke-Alzheimer’s Disease and Related Disorders Association working committee and the National Institute of Neurological and Communicative Disorders both accepted the term “probable AD.” In Alzheimer’s disease, the limbic lobe structures in the brain often exhibit significant shrinkage, while the frontal and temporal cortices commonly show enlargement of the ventricles and atrophy of the gyri. However, the major motor and somatosensory cortices are typically unaffected in most AD patients. The precuneus and posterior cingulate gyrus have been extensively studied in functional imaging investigations. Frequently, medial temporal atrophy which impacts the amygdala and hippocampus, is associated with temporal horn expansion and a reduction in brain weight (Sengoku, 2020). We utilized DEG detection, GO and KEGG analysis, PPI network construction, and hub genes identification to integrate multiple datasets and identify functional genes associated with AD. Furthermore, we also identified miRNAs that target the hub genes.

The *SELE* (*Selectin-E*) gene is responsible for producing E-selectin, which is found in cytokine-stimulated endothelial cells and play a critical role in promoting the accumulation of leukocytes in inflamed areas by mediating their adhesion to the vascular wall. E-selectin has a complex structure, consisting of lectin- and EGF-like domains followed by short consensus repeat domains with six conserved cysteine residues. E-selectin is classified as a cell adhesion molecule belonging to the selectin family of glycoprotein sugar chains or sheath sugars that are present on the surface of white blood cells (Wang et al., 2020). This molecule is believed to be accountable for facilitating the adhesion of leukocytes to the vascular linings, thus, enabling their accumulation at sites of inflammation. According to Li et al. (2015), E-selectin levels in CSF are considerably higher in clinically diagnosed AD patients who did not have the usual AD CSF biomarker signature (i.e., low tau/Aβ_42_ ratio) compared to those who did have a positive biomarker signature. *SELP* (*Selectin P*), another gene in this family, was found to be overexpressed in AD patients with fast cognitive decline and therefore, proposed as a prognostic biomarker and a potential target for treatment in a previous study (Stellos et al., 2010). Hence, studying the baseline of E-selectin in AD patients may result in finding novel biomarkers.

*TGF-β* genes (*Transforming growth factor beta genes*) are pleiotropic cytokines that orchestrate numerous key physiological processes including as embryogenesis, immunological response, extracellular matrix metabolism, and cell circle formation. Indeed, *TGF-βs* are a subgroup of a larger family of more than 40 structurally similar regulatory proteins expressed in mammals, which includes bone morphogenetic proteins (BMPs), growth and differentiation factors (GDFs), Mullerian inhibitory factor (MIF), activins, and inhibins (Zhang et al., 2016). *TGF-β1* stimulates the formation of amyloid precursor protein and subsequent amyloid-beta generation in murine and human astrocyte cultures, and transgenic animals overexpressing *TGF-β1* in astrocytes cause amyloid-beta deposition. Furthermore, post-mortem brain examinations of AD patients indicate elevated *TGF-β1* expression (Zetterberg et al., 2004). According to Wyss-Coray et al. (2001) *TGF-β1* has been linked to enhanced a clearance from the brain parenchyma by activated microglia cells and decreased amyloid-beta plaque formation in aged transgenic mice expressing the human amyloid precursor protein. It is worth noting that *TGF-β1* plays a vital role in decreasing inflammation and possesses protective effects on the brain. This growth factor affects the brain’s functioning by utilizing a signaling pathway that involves SMAD proteins. Abnormalities in the TGF-β1/SMAD pathways, which are activated by *TGF-β* and act as transcription factors within the cell, have been detected in relation to Alzheimer’s disease (AD) (Yang and Xu, 2023).

*MYH11* (*myosin heavy chain 11*) is a component of a hexameric protein composed of two heavy chain subunits and two pairs of non-identical light chain subunits. *MYH11* has been extensively studied for its potential involvement in vascular disease and stroke which are associated with AD (de la Torre, 2006; Kuang et al., 2012). Missense mutations in MYH11 have been linked to higher levels of insulin-like growth factor-1 (IGF-1), angiotensin-converting enzyme (ACE), and macrophage inflammatory protein-1α and *β* (MIP-1-α and MIP-1-β) expression in the aorta and explanted aortic smooth muscle cells of a patient with non-syndromic thoracic aortic aneurysms and dissections (Pannu et al., 2007). The contribution of *MYH11* to AD through TGF-β pathway has not been studied yet. Besides, the association between the mutations in this gene and TGF-β pathway is controversial. Renard et al. (2013) discovered that an in-frame splice-site mutation in the *MYH11* gene was related to higher levels of the TGF-β pathway activity in individuals with non-syndromic familial thoracic aortic aneurysms. Pannu et al. (2007), on the other hand, found no link between missense mutations in the *MYH11* gene and an upregulation in TGF-β pathway activity in patients with non-syndromic thoracic aortic aneurysms and dissections. In general, most research has indicated that *MYH11* can contribute to AD by promoting vascular disease and upregulating the TGF-β pathway.

*ITGB1* (*Integrin β1*) is a well-known integrin heterodimer sub chain. *ITGB1* bidirectional signaling, as well as cross-talk with other cellular receptors, has been demonstrated to be critical in survival, cell adhesion, differentiation, and proliferation (Miranti and Brugge, 2002). It also enhances tumor treatment resistance and is required for the survival and metastatic potential of lung, breast, and colon tumors (Jiang et al., 2021). Pan et al. (2011) discovered that when microglial cells are exposed to oligomeric amyloid-beta or an inflammatory and oxidative environment, the mRNA expression of the *ITGB1* receptor is reduced. This reduction in *ITGB1* expression may contribute to AD-related poor phagocytosis and clearance of amyloid-beta fibrils. Another gene in this family, *ITGB3*, (or platelet glycoprotein IIIa), was shown to be overexpressed in Alzheimer’s disease patients with rapid decline in cognition. Stellos et al. (2010) examined the basal level of activated glycoprotein IIb-IIIa complex in a 1-year study of Alzheimer’s disease patients. Patients with fast cognitive decline had considerably greater expression baseline than those with slow cognitive decline. As a result, they proposed that Gp IIb-IIIa might be a possible biomarker for the pace of cognitive decline and a potential novel therapy target in AD patients. Thus, baseline *ITGB1* expression might be a potential biomarker and a therapy target for AD.

*CXCR4* (*CXC motif chemokine receptor type 4*) is a strongly conserved chemokine that is essential for stem cell trafficking, lung repair, and cancer metastasis (Strieter et al., 2006). Studies have suggested that the *CXCL12/CXCR4* signaling pathway is implicated in the pathologic process which is important for influencing numerous nervous system developmental processes as well as regulating synaptic plasticity. *CXCR4* is thought to contribute to the formation and progression of AD by influencing clearing of amyloid-beta plaques and the immune cell migration in the brain (Li and Wang, 2017).

Additionally, as a member of the G-protein-coupled receptor (GPCR) family, *CXCR4* plays a crucial role in cell migration, neurotransmission, and inflammatory responses within the central nervous system (CNS). In the context of AD, *CXCR4* has been implicated in promoting inflammation mediated by microglia through pathways such as JAK/STAT and NF-κB. Interestingly, *CXCR4* may also exhibit protective effects on neurons (Li and Wang, 2017). Moreover, the activation of *CXCR4* in astrocytes, another type of CNS cell, can modulate neurotransmission and contribute to the intricate interactions between glial cells and neurons in AD. While the precise mechanisms by which *CXCR4* influences AD progression require further investigation, targeting *CXCR4* shows promise as a potential therapeutic approach for AD. Gaining a deeper understanding of the role of *CXCR4* in AD has the potential to inspire innovative strategies for the prevention, diagnosis, and treatment of this debilitating disease (Wang et al., 2022).

MicroRNAs are complex non-coding RNAs that play a pivotal role in modulating numerous cellular functions (Wang et al., 2009). Due to miRNAs involvement in various biological pathways, they’re aberrant expression may be linked to the pathogenesis of numerous disorders. Hence, obtaining a comprehensive understanding of the vital mechanisms underlying the interaction between miRNAs and their targets could offer valuable insights into the pathogenesis of neurodegenerative diseases such as AD (Yang et al., 2022).

The current study investigated 73 differentially expressed miRNAs (DEmiRS), through meta-analyzing a single miRNA dataset. Notably, *hsa-miR-601* displayed the highest LogFC and its upregulation is believed to be correlated with specific developmental stages particularly in modulating actin-cytoskeleton. It is worth noting that the disruption in actin-cytoskeleton dynamics might be a contributing factor to synaptic disfunction, observed in AD (Bamburg and Bloom, 2009; Ohdaira et al., 2009). Moreover, *hsa-miR-374* showed the lowest LogFC among all analyzed miRNAs which has been implicated in neural differentiation, however, further research is needed to elucidate its precise role in AD (Sun et al., 2022).

Additionally, we examined the correlation between miRNAs and hub genes, in which our results revealed that *hsa-mir-122-5p* is a critical miRNA closely associated with four hub genes (*TGFB1, ITGB1, CXCR4, and MYH11*). Although little is known about the exact molecular mechanism underlying the pathogenesis of this miRNA in AD, its downregulation in Alzheimer’s diseases patients has been confirmed as a potential new miRNA candidate (Kumar et al., 2017). In contrast, a significant increase in *miR-122–5p* expression has been observed in AD patients as compared to healthy controls (Guévremont et al., 2022).

*hsa-mir-3200-3p* is another miRNA which is closely associated with three hub genes (*TGFB1, ITGB1, CXCR4*). In the context of Alzheimer’s disease, mir-3200-5p was found to be notably downregulated, which has been implicated in neural synaptic functions (Satoh et al., 2015). Furthermore, recent investigations have demonstrated that *hsa-mir-106a-5p* which based on our results is linked to (*TGFB1, ITGB1, and CXCR4),* reduces the level of VEGFA, a protective factor against cognitive impairment in AD patients. As a result, decreased levels of this miRNA may potentially be associated with cognitive impairment in AD (Abyadeh et al., 2022).

Moreover, recent human trials have demonstrated that both miRNAs hsa-miR-9-3p and hsa-miR-9-5p play role in the delicate balance between *BACE1* and Aβ peptides. The study by Sun et al. (2023) have proven that hsa-miR-9-3p which based on our findings is associated with *(ITGB1, CXCR4, and SELE),* target *BACE1* which contributes to the elevation Aβ_42_ and aggravate AD. Besides, hsa-miR-9-3p has a negative regulatory effect on the expression of *GDF11* (growth differentiation factor 11), a member of the TGF-β superfamily which was previously discussed. All monomeric forms of Aβ, including Aβ_42_, are produced by the enzyme *BACE1* (β-site amyloid precursor protein cleaving enzyme 1). The concept that *BACE1* might encourage AD development is supported by the fact that *BACE1* levels and activity rates are higher in AD brains and body fluids (Hampel et al., 2021).

Based on our findings, *hsa-mir-21-5p* was identified as a miRNA associated with two hub genes, namely, *TGFB1* and *MYH11.* Previous studies have reported significant downregulation of this miRNA in AD patients. Considering that the associated hub genes are involved in SMAD protein phosphorylation, it is possible that this miRNA play a vital role in regulating these proteins, as well (Burgos et al., 2014). SMAD proteins are known to be important in AD, as they have been detected within amyloid plaques and neurofibrillary tangles, which are pathological hallmarks of AD. Thus, Gámez-Valero et al. (2019) suggested that *hsa-mir-21-5p* could potentially be involved in the development of AD.

We also have identified several other miRNAs that interact with common hub genes, suggesting their substantial roles in the initiation or progression of AD, including *has-mir-27a-3p (TGFB1, ITGB1, and CXCR4), hsa-mir29b-3p (ITGB1, CXCR4, and SELE), hsa-mir-130a-3p (MYH11, TGFB1, and CXCR4),* and *hsa-mir-17-3p (SELE and ITGB1).* These findings highlight the potential significance of miRNAs, providing insights into the molecular mechanisms underlying AD pathogenesis.

In conclusion, our meta-analysis employing accessible datasets was able to clarify and identify putative biomarkers related to AD. As a result, the identification of five prospective gene biomarkers that are differently expressed in AD, such as *SELE, TGFβ1, MYH11, ITGB1,* and *CXCR4*, offers possibilities for clinically diagnostic biomarkers that can identify AD patients. Furthermore, protein interaction research revealed major pathways linked to probable biological events such as various dysregulated biochemical pathways (actin cytoskeleton, ECM-receptor interaction, endocytic vesicle dysfunction, hypertrophic cardiomyopathy, and TGF-β pathway). Validation of these biomarkers and metabolic pathways by experimental and functional research might add to the current study’s findings.

## Data Availability

The authors acknowledge that the data presented in this study must be deposited and made publicly available in an acceptable repository, prior to publication. Frontiers cannot accept a manuscript that does not adhere to our open data policies.
